# Odontogenic Keratocyst Mimicking Paradental Cyst

**DOI:** 10.1155/2014/974241

**Published:** 2014-07-08

**Authors:** Andrea Enrico Borgonovo, Luigi Bernardini, Paola Francinetti, Federica Rizza, Dino Re

**Affiliations:** ^1^Department of Oral Surgery, Fondazione IRCCS Ca' Granda, Ospedale Maggiore Policlinico, Via Commenda 10, 20122 Milan, Italy; ^2^Department of Oral Rehabilitation, Istituto Stomatologico Italiano, University of Milan, Via Pace 21, 20122 Milan, Italy

## Abstract

*Objective*. The aim of this paper is to present an uncommon clinical and radiographic aspect of odontogenic keratocyst (OKC) mimicking paradental cyst. *Methods*. A 32-year-old female patient showed a well-delimited radiolucent lesion connected with the root of the left third molar with close anatomical relationship with the mandibular canal. The clinical, radiographic, and anamnestic features lead us to diagnose a paradental cyst that was treated by enucleation after extraction of the partially impacted tooth. *Results*. Histological analysis showed typical histological features of PKC such as the presence of a lining of stratified squamous epithelium with a well-defined basal layer of palisading columnar of cuboidal cells. *Conclusion*. Initial X-ray analysis and the position of the lesion related to the third mandibular tooth caused us to mistakenly diagnose a paradental cyst. We were only able to identify the cyst as an PKC rather than a paradental cyst after histological analysis.

## 1. Introduction

Odontogenic keratocyst (OKC) was categorised as a form of odontogenic tumor in 2005 by a WHO working group. WHO defined OKC as “a benign multicystic, intraosseous tumor of odontogenic origin, with a characteristic lining of parakeratinized stratified squamous epithelium and potential for aggressive, infiltrative behavior” [1].

Malignant transformation into squamous cell carcinoma is unusual. There exists a second variant of OKC, the orthokeratocyst, which is less aggressive and occurs less frequently (12% of OKC) but is still classified as an odontogenic cyst [2].

The odontogenic keratocyst is often associated with Gorlin-Goltz syndrome and is characterised by the presence of multiple nevoid basal cell epitheliomas, multiple keratocysts in the jaws, and bifid ribs [3]. The incidence of this syndrome is in the order of 1 in 57,000 to 256,000 in the general population [4].

The most common clinical symptoms are swelling, discharge, and pain, but in some cases OKC may be asymptomatic: the frequency of a casual diagnosis ranged between 5,5% and 42,5%.

The aim of this paper is to present uncommon clinical and radiographic features of OKC mimicking paradental cyst.

## 2. Case Presentation

**Figure 1:**
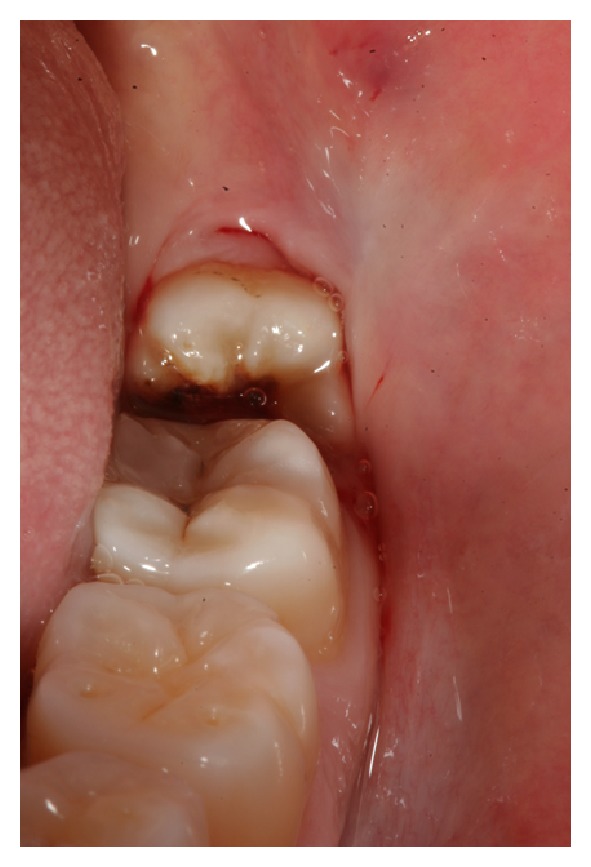
Preoperative clinical view.

A 32-year-old female patient visited our clinic for a routine control. Clinical examination of the oral cavity revealed the presence of two partially impacted third mandibular molars. They did not show clinical aspects of inflammation such as swelling or pain and the mucosa around the tooth site appeared clinically normal (Figure 1).

**Figure 2:**
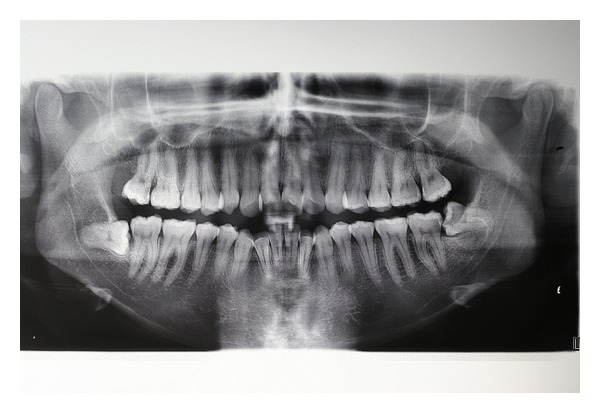
Preoperative orthopantomography.

A panoramic radiograph (OPG) revealed a well-defined radiolucency associated with the left third molar (Figure 2), prompting us to prescribe a computed tomography to better investigate the lesion.

**Figure 3:**
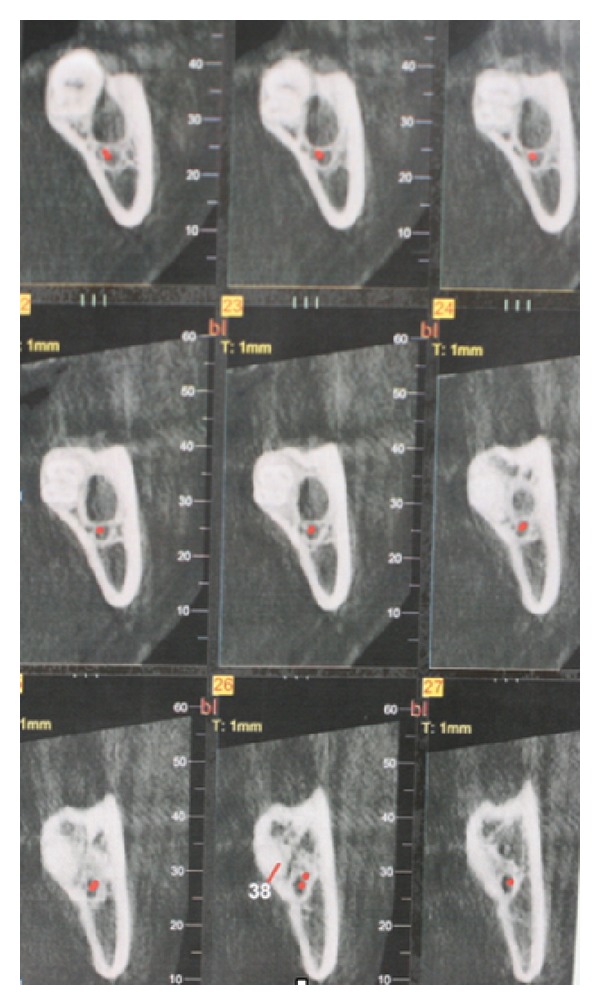
Preoperative tomography.

Computed tomography evidenced a well-delimited radiolucent lesion connected with the root of the left third molar and confirmed a close anatomical relationship between the cyst and the mandibular canal (Figure 3). The clinical, radiographic, and anamnestic features lead us to diagnose a paradental cyst.

**Figure 4:**
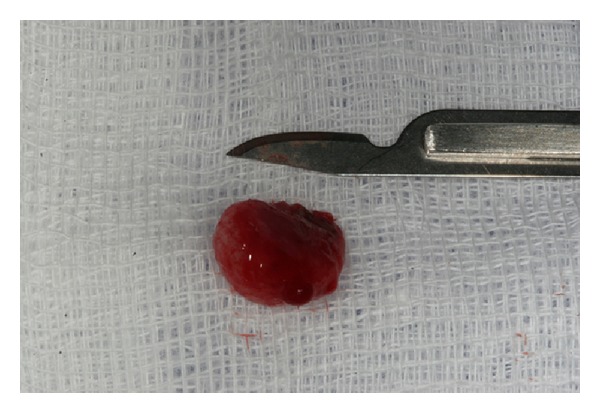
Cyst enucleation.

The partially impacted tooth was removed surgically under local anesthesia and the healing suffered no complications. Our surgical approach involved a trapezoidal flap with vestibular ostectomy, and the cyst was treated by enucleation (Figure 4). The wound was irrigated with sterile saline and a suture was made with silk 4/0.

**Figure 5:**
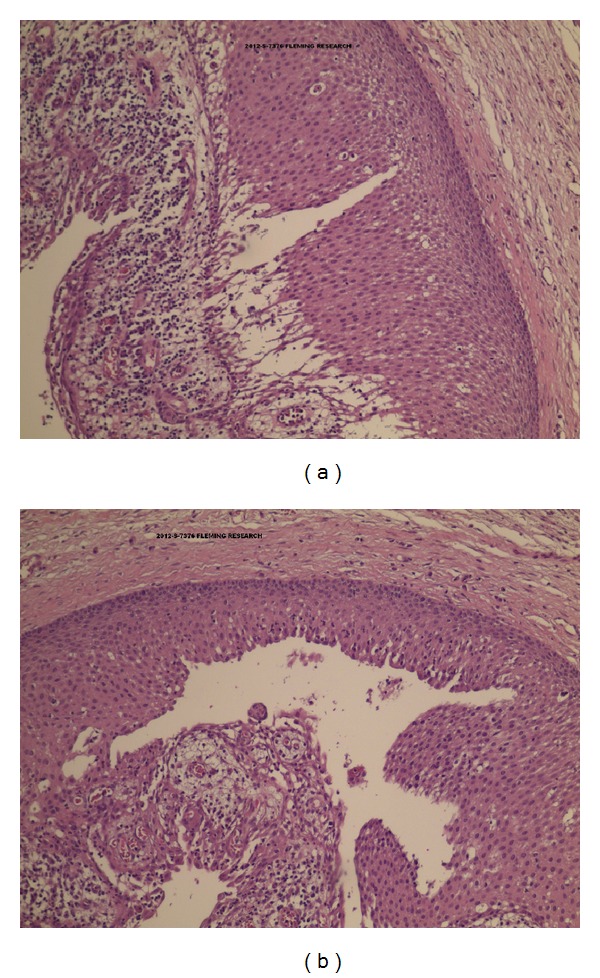
Histological pictures.

Histological analysis showed typical histological features of PKC such as the presence of a lining of stratified squamous epithelium with a well-defined basal layer of palisading columnar of cuboidal cells (Figure 5).

These histological results determined our choice of follow-up program, which was selected to control the aggressive behaviour of PKC.

Our protocol for patients with PKC includes clinical and radiographic examinations every three months for the first year after surgery and once a year from then on with a TC control every two years.

**Figure 6:**
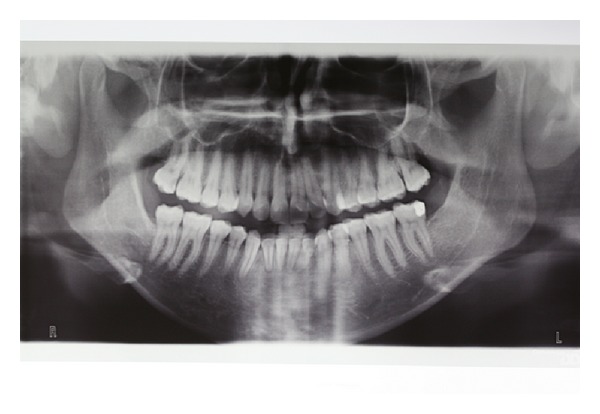
Orthopantomography at 1 year.

The orthopantomography conducted one year after surgery showed no signs of relapse (Figure 6).

## 3. Discussion

Differential diagnosis represents an important and complex phase of the clinical process. Diagnosis can often be no more than hypothetical, despite thorough anamnesis, accurate clinical analysis, and instrumental examination.

A radiolucent lesion, located in the mandibular area, can be more or less aggressive in nature (myxoma, ameloblastoma, keratocyst, etc.). Differential diagnosis must therefore be executed in order to accurately identify the lesion.

In the clinical case under examination, the lesion was small and unilocular, presented clearly defined edges, was not corrosive, and was located in proximity of the mandibular angle, associated with the third molar in dysodontiasis. The lesion was classified as a paradental cyst.

This particular classification was diagnosed due to the close proximity of the lesion to the root of the third molar. Follicular cyst and OKC were therefore excluded. Histological examination of the biopsy, however, contradicted this diagnosis, indicating a case of PKC.

Diagnosis of OKC is highly important due to its aggressive behaviour and the frequency of recurrence after surgical treatment, which has been observed to occur at a rate between 2,5% and 62,5% [5, 6] and up to 82% when associated with NBCCS [7]. Possible explanations for this high frequency of recurrence are a large amount of fibrinolytic activity in the cyst wall [8], increased mitotic activity [9], epithelial proliferation in connective tissue, and residual dental lamina with subsequent formation of new cysts [10].

Diagnosis of OKC is often very difficult because its clinical and radiographic aspects are aspecific. Common radiographic features are unilocular or multilocular well-circumscribed radiolucent lesions surrounded by a thin sclerotic border. While the multilocular variant is difficult to differentiate from other odontogenic or nonodontogenic neoplasms like ameloblastoma [11], the unilocular variant is difficult to distinguish from other odontogenic or nonodontogenic cysts like periapical cysts, dentigerous cysts, lateral periodontal cysts, or paradental cysts. The most common site of involvement is the mandible, where OKC appears in approximately 75% of cases, in particular within the body, angle, and ramus region, while in the remaining 25% of cases it appears within the maxilla [7]. OKC is frequently located within the periapical region of mandibular teeth, can envelope the crowns of unerupted teeth, or can be located between the roots of the teeth, making it particularly difficult to distinguish from other cysts. Paradental cysts in adults can be particularly difficult to distinguish from OKC as they are localized exclusively in the mandibular region related to the third molars, while the juvenile one is often related to the second [12] and first molars [13] and rarely in premolars [14] or incisors/canines [15]. Over 60% of the cases of paradental cysts are associated with the lower third molars [16] and are normally characterized by a unilateral presentation but in the literature some cases of bilateral occurrence are reported [13].

From a clinical perspective, these lesions can demonstrate similar symptoms such as swelling, pain, or, in some cases, suppuration from pericoronal sulcus [17–21]. In certain cases they may be asymptomatic [22] and discovered accidentally during routines controls. In order to differentiate an OKC from a paradental cyst with absolute certainty, a histological analysis must be made after surgical excision.

In our case, initial X-ray analysis and the position of the lesion related to the third mandibular tooth caused us to mistakenly diagnose a paradental cyst. We were only able to identify the cyst as an OKC rather than a paradental cyst after histological analysis. From a histological perspective, there are marked differences between the two types of cyst. OKC is characterised by the presence of a thin bandlike parakeratinized stratified squamous epithelium, with a prominent basal layer composed of palisade-like columnar cells and a connective tissue wall that generally does not demonstrate inflammation. Paradental cysts, like other inflammatory cysts, present chronic inflammatory cell infiltration and are lined by a nonkeratinised stratified squamous epithelium [17].
